# The coding of telephone consultations in UK primary care databases: are we picking up all the calls?

**DOI:** 10.1186/s13104-023-06325-y

**Published:** 2023-05-09

**Authors:** Mark D Atkinson, Roxanne Cooksey, Jenna K Jones, Sinead Brophy

**Affiliations:** 1 National Centre for Population Health and Wellbeing Research, Swansea University Medical School, Swansea, Wales; 2 Swansea University Medical School, Swansea, Wales

**Keywords:** Telephone consultation, General practitioner, Electronic health record

## Abstract

**Objectives:**

To examine the use of two coding systems used in the THIN UK primary care research database for the coding of telephone encounters between patient and healthcare professional in primary care. This is relevant to other research databases built on GP clinical systems. Consideration of telephone consultations was particularly important during the COVID-19 pandemic as remote interactions between patient and GP are more numerous than before and are likely to remain at a higher frequency.

**Results:**

Telephone encounters could either be indicated by a consultation-type code or by a Read code. All three possible combinations (coded by one method, the other method and both) were in use. In 2014, 30% were coded by the consultation-type, 55% by Read codes and 15% by both. In contrast, in 2000, 77% were coded by the consultation-type, 21% by Read codes and 2% by both. This has important implications because national and regional consultation rates by GPs are often estimated from these research databases by looking only at the consultation-type codes and consequently many encounters will not be detected.

## Introduction

Telephone calls are one mode of consultations with patients. These have recently had a sharp increase during the Covid-19 crisis and it is important to be able to monitor the use of this type of consultation over time. At the present time (during and after the COVID-19 pandemic), the telephone call with a patient, both for triage and the consultation, is having a considerable resurgence and may well continue at a higher level into the future. In many countries, primary care practitioners have increased the use of telephone, email and virtual consulting [1]. Both telephone and video consultations have led to great time savings in some UK practices [2] and this seems likely to persist in the future. It may be that remote consultations (both by telephone and by video conferencing) could become a more frequent modality for consultations. With changes in demand for GP services over time, the monitoring of consultation rates in general, and different types of consultation is instrumental in informing the provision of effective GP services [3]. Here we examine the coding of telephone consultations in UK general practice by looking at one clinical computer system, Vision (In Practice Systems Limited). Vision is used by GPs during the course of a consultation with a patient. There are two research databases which provide extracts of the Vision system from some GP practices to researchers. These are The Health Improvement Network (THIN) and Clinical Practice Research Datalink (CPRD). We use an extract of the THIN database made in 2015 to show how the coding of these consultations is done and to highlight some complexities in estimating numbers of consultations. Because of the date of this extract, we cannot use it to demonstrate the current upsurge in numbers of telephone consultations, but we examine the coding complexities which are still present.

## Main text

### Coding of telephone consultations

The Vision clinical database is organised into a number of files (Medical, Therapy and Additional health data). The unit of recording is the consultation, each of which has an Identification (ID) number and may have entries in any of the three files and is also associated with a type of consultation, which has 61 possible values referring to the type of consultation and a staff type (GP, nurse, etc.).

Within a consultation there can be one or many events, each coded by a Read code, which are used throughout the system to code the whole range of events of clinical interest; diagnoses, symptoms, treatment, prescriptions, patient information, test results and administrative functions [4].

Telephone consultations can be coded with 4 possible consultation type values (Table 1). However, the complexity referred to earlier arises from the use of Read codes which can also be used to indicate a telephone call to or from a patient (Table 2). Therefore we see there are effectively two systems for coding telephone consultations.

**Table 1:** The four values of the LOCATE code used for telephone consultations. Percentages are given of the total instances of all 4 codes

Code	Description	Percentage
U	Telephone call to a patient	60.7
J	Telephone call from a patient	39.3
g	Telephone consultation	< 0.01
f	Co-op telephone advice (out of hours)	< 0.01

**Table 2:** Read codes referring to a telephone call between patient and medical professional, without a specialised purpose

Read code	Description	Percentage of total instances
9N31.	Telephone encounter	87
9N3A.	Telephone triage encounter	8
8CAN.	Patient given telephone advice during surgery hours	2.9
8CAK.	Patient given telephone advice out of hours	0.9
9N3F.	Nurse telephone triage	0.2
8H9.	Planned telephone contact	0.2
9Nj0.	Unsuccessful attempt to contact patient by telephone	0.2
9b0m.	Telephone call from a patient	0.01
9b0n.	Telephone call to a patient	0.03
9b0o.	Telephone conversation	0.03
9b0m.	Telephone call from a patient	0.02
9N311	Telephone follow-up	0.01

Each GP will have their own approach to data entry which may be influenced by their training or local practice and here we are guided by one of the authors (JKJ). At the start of a consultation, the GP will select a consultation type code. Each surgery will select a default, often ‘surgery consultation’, and this may be chosen for a telephone consultation if the GP is short of time or forgets to set the value explicitly. When making a triage call initially, the GP may select ‘telephone call to a patient’ as the consultation type. The GP may keep the locate code as surgery consultation and so there will be two separate consultations, one on the phone initially, and then one face to face. In these cases, the Read codes used are usually ‘Telephone encounter’ (9N31.) and then ‘Patient reviewed’ (6 A…). If a telephone call to a patient is about results, referral updates and other matters concerning existing problems, the administration consultation type may be used. If the patient cannot be reached on the telephone the Read code ‘Failed encounter’ (9N4.) may be used.

### Data

An extract of patients in all Welsh GP practices contributing to the THIN database with rheumatoid arthritis was requested for a study on whether disease flares were associated with seasonal influenza vaccination. This required the estimation by various means, of the numbers of contacts made by patients with their GP both before and after vaccination, and those made by unvaccinated patients. This extract had data from 3321 patients and was approved as 14THIN063 by the THIN Scientific Review Committee and was made in 2015. The data will be deleted, in accordance with the access agreement when the work is completed. In the THIN database extract, the variable coding for consultation type is called LOCATE.

## Results

The LOCATE variable has four values for telephone consultations (Table 1). The value U (Telephone call to a patient) is used about twice as often as the value J (Telephone consultation from a patient) (60.7% vs. 39.3%) and the other two are used very rarely (both < 0.01%), so we exclude these from further analysis. There are many Read codes which indicate that a telephone call took place. Many of these are for very specialised uses of the telephone which are included here for completeness. There are 57 codes for invitations and in general these are not widely used. One which is used is 9OX7. (Influenza vaccination telephone invite). For all uses of these Read codes, only 3% are associated with one of the LOCATE values associated with telephone consultations (Table 1). The other 97% are associated with 6 other LOCATE values, the main one of which, with 28% of the instances is S (Administration) and the second, with 9% is O (Other). Most of these types of calls are more likely to be made by nurses or administrative staff than by doctors. However, there are 11 codes (Table 2) which show that a telephone call without a specialised purpose was made, and it is clear that the code 9N31. (Telephone encounter) is by far the most frequently used. Note also that the presence of this code this does not indicate the direction of the call. We have included all 11 of these codes in the analysis because we expect Read codes in other extracts could be in different proportions. With these 11 codes, the most frequent associated LOCATE value is I (Surgery Consultation), the second most frequent is S (Administration) and the third and fourth most frequent are U and J. This emphasises that Read codes indicating telephone consultations are most frequently associated with LOCATE codes which do not. We make an assumption throughout this work that if a consultation is coded either by the LOCATE variable or by a Read code as a telephone consultation, then we consider it to be a telephone consultation.

So it is evident that a consultation in which a telephone contact between patient and healthcare professional is made, can be coded either by a relevant Read code or by a relevant LOCATE value, or by both (Table 3). There is a trend over the years from 2000 to 2014 in which the use of the LOCATE codes U and J only has declined from around 78% to around 30%. In these cases this was the only evidence of a telephone consultation. In parallel, those coded by the group of eleven Read codes has risen over the same period from 20 to 55% and similarly for the use of both coding systems, there has been a rise from 3 to 15%.

**Table 3:** The three final columns are the percentages number of telephone consultations detected by the two coding methods alone or both in combination. U and J refer to the LOCATE values

		% of total		
Year	Total	U/J only	Read codes only	Both
2000	993	76.7	20.7	2.5
2001	1191	78.7	16.7	4.6
2002	1394	64.9	28.6	6.5
2003	2102	70.7	22.5	6.8
2004	3232	59.3	28.2	12.4
2005	4139	57.6	30.1	12.3
2006	3831	45.8	41.1	13.1
2007	4381	39.2	47.1	13.7
2008	4711	33.2	51.4	15.4
2009	5133	32.2	50.7	17.1
2010	5648	28.1	54.6	17.4
2011	6178	28.5	53.5	18
2012	7152	28.8	53.7	17.5
2013	7194	27.1	55.7	17.1
2014	2206	29.5	55.2	15.4

## Discussion

In this paper, we are concerned with the recording of the occurrence of a telephone communication between a patient and a medical professional. We are interested in how the two coding systems complement each other. These clinical databases are flexible and can be used in many ways, reflecting the complexity and pressures of the consultation process.

Our original reason for looking at this question was to examine different ways to quantify the number of contacts with GPs made by patients and a close examination revealed the two parallel but non-identical systems. If the frequency of telephone consultations over a period of time is estimated by counting instances of relevant LOCATE codes, many consultations will be missed (Table 3). Moreover, but perhaps of less interest, the vast majority of special calls for invitations will not be counted.

An important consequence of our findings arises because most studies which examine numbers of consultations of different types use the consultation type code alone [3, 5]. Clearly there will be some underestimation as a result of this. The solution to this is simply to take both Read codes and LOCATE types into account. It is difficult to know why the proportions of telephone consultations detected by Read codes has risen, but it would be premature to speculate until more recent samples have been examined.

Similar situations are present in the coding of other consultation types, although the principal LOCATE code for a GP consultation does not have any consistent use of Read codes describing a consultation.

As explained above, this work was carried out with data from a particular clinical system in which the research database keeps, at least in part, some of the complexity of the system as used in clinic. Other primary care research databases as for example the SAIL databank [6, 7], only include the Read codes, dates and values from the contributing clinical databases. These, therefore, do not have the LOCATE codes associated with each consultation, and rely on the use of Read codes alone, and again will underestimate the true numbers of telephone consultations, but no obvious solution to this is evident.

This study provides a useful exploration of the coding practices of telephone encounters pre-COVID-19. Our findings highlight that using consultation-type codes alone may miss the true number of consultations causing an underestimation.

## Limitations

This study is based on data from relatively small number of patients from a small geographical area, having a particular chronic condition and stored in a particular clinical system. However, the findings concern coding practice and are unlikely to be applicable only in this group. Nevertheless, a larger survey is needed to see if there are differences between practices, health boards and regions and to ascertain the temporal trend with more precision, particularly by including more recent data.

## Data Availability

The data were made available by application to the data owner and cannot be transferred.

## Abbreviations

CPRD: Clinical Practice Research Datalink
GP: General Practitioner
SAIL: Secure Anonymised Information Linkage
THIN: The Health Information Network
